# Experimental data for green synthesis of Zn-abietate complex from natural resin

**DOI:** 10.1016/j.dib.2021.107776

**Published:** 2021-12-31

**Authors:** Jamille S. Correa, Júlia O. Primo, Carla Bittencourt, Dienifer F.L. Horsth, Eduardo Radovanovic, Alceu T. Silveira-Jr, Henrique E. Toma, Cristina M. Zanette, Fauze J. Anaissi

**Affiliations:** a Departament of Chemistry, Universidade Estadual do Centro-Oeste, UNICENTRO, Alameda Elio Antonio Dalla Vecchia, 838, Guarapuava, PR 85040-167, Brazil; b Department of Chemistry, University of Mons, Place du Parc 23, Mons 7000, Belgium; c Department of Chemistry, Universidade Estadual de Maringá, UEM, Av. Colombo 5790, Maringá, PR 87020-900, Brazil; d Institute of Chemistry, University of Sao Paulo, São Paulo, SP 05508.000, Brazil

**Keywords:** *Pinus elliottii* resin, Abietic acid, Mass spectra, XPS, EDS

## Abstract

This data article is associated with the work “Ecofriendly synthesis of Zn-abietate complex derived from *Pinus elliottii* resin and its application as an antibacterial pigment against *S. aureus* and *E. coli*”. The characterization data of the Zn-abietate complex obtained from *Pinus elliottii* resin and their reactional intermediary (Na-abietate) are reported. The Na-abietate was prepared with purified Pinus resin and sodium hydroxide (≥ 99%) in a stoichiometric ratio of 1:1. For the Zn-abietate synthesis was used ZnSO_4_ and Na-abietate solutions were at mild temperature and stirring without using organic solvents to ensuring the green character of the synthesis. Spectroscopic and structural characterization was consistent with an octahedral complex involving three carboxylate ligands per metal ion. X-ray photoelectron spectroscopy (XPS) analysis of the Na-abietate salt confirms the presence of carbonyl groups, carbon-oxygen atoms simple bonds (O-C/O=C), and carboxylate groups oxygen atoms (O-C=O). Analysis of the Zn LMM Auger, for the Zn-abietate complex, indicates the presence of zinc atoms with oxidation state Zn^2+^, this is supported by the distance between Zn 2p_3/2_ and 1p_1/2_ in the XPS spectrum. Together, these data will be useful for the structural representation of the samples.

## Specifications Table

**Table:** 

Subject	Inorganic Chemistry
Specific subject area	Science Materials
Type of data	Figure and table
How the data were acquired	The mass spectra (MS) Bruker Amazon Speed ETD equipment, ion trap (MS-MS) with low resolution, in negative ion and ionization by electrospray mode. A drying gas flow of 4 L min^−1^, at a temperature of 200 °C, nitrogen as a nebulizer gas under pressure of 7 psi, and a voltage of 4500 V. X-ray photoelectron (XPS) spectroscopy (Versaprobe PHI 5,000 from Physical Electronics, equipped with a monochrome X-ray source Al Kα). The XPS spectra were collected at a take-off angle of 45^o^ in relation to the energy of the analyser electron and the spot size was 200 µm. Passage energy (PE) of 20 eV was used for the high energy resolution spectra (Zn 2p, Na 1s, O 1s, and C 1s). The energy resolution was 0.6 eV. For the charge compensation of build-up charge on the sample surface during measurements, a dual beam charge neutralization composed of an electron gun (< 1eV) and an argon ion gun (< 10 eV) was used. The XPS spectra were analysed using the CASA-XPS software.
Data format	Raw and analysed
Description of data collection	The mass spectra (MS) of Zn-abietate were obtained from a solution of dichloromethane (DCM) diluted in methanol. For the XPS experiments, the samples were pressed to form 1 mm thick pallets, the pallets were supported on UHV compatible double-sided tapes attached to a customized sample holder.
Data source location	Universidade Estadual do Centro-Oeste, Guarapuava, Brazil. University of São Paulo, São Paulo, Brazil. Universidade Estadual de Maringá, Maringá, Brazil University of Mons, Mons, Belgium. And DOI: 10.17632/n4fhxhz77b.1
Data accessibility	The raw data is found in Mendeley repository: DOI: 10.17632/n4fhxhz77b.1 https://data.mendeley.com/datasets/n4fhxhz77b/1
Related research article	J.S. Correa, J.O. Primo, C. Bittencourt, D.F.L. Horsth, E. Radovanovic, A.T. Silveira-Jr, H.E. Toma, C.M. Zanette, F.J. Anaissi. Ecofriendly synthesis of Zn-abietate complex derived from Pinus elliottii resin and its application as an antibacterial pigment against S. aureus and E. coli. Dyes and Pigments, 197 (2022) 109946. 10.1016/j.dyepig.2021.109946

## Value of the Data

• A green, simple, low-cost method to produced zinc abietate. Data obtained show the ligand coordination, the chemical species present on the surface, and the chemical composition of the samples.
• The method can be useful to avoid using organic solvents and the data can contribute to identifying components and structure.
• Data can be used to determine the structure and properties of the materials and the metals.

## 1 Data Description

**Fig. 1:**
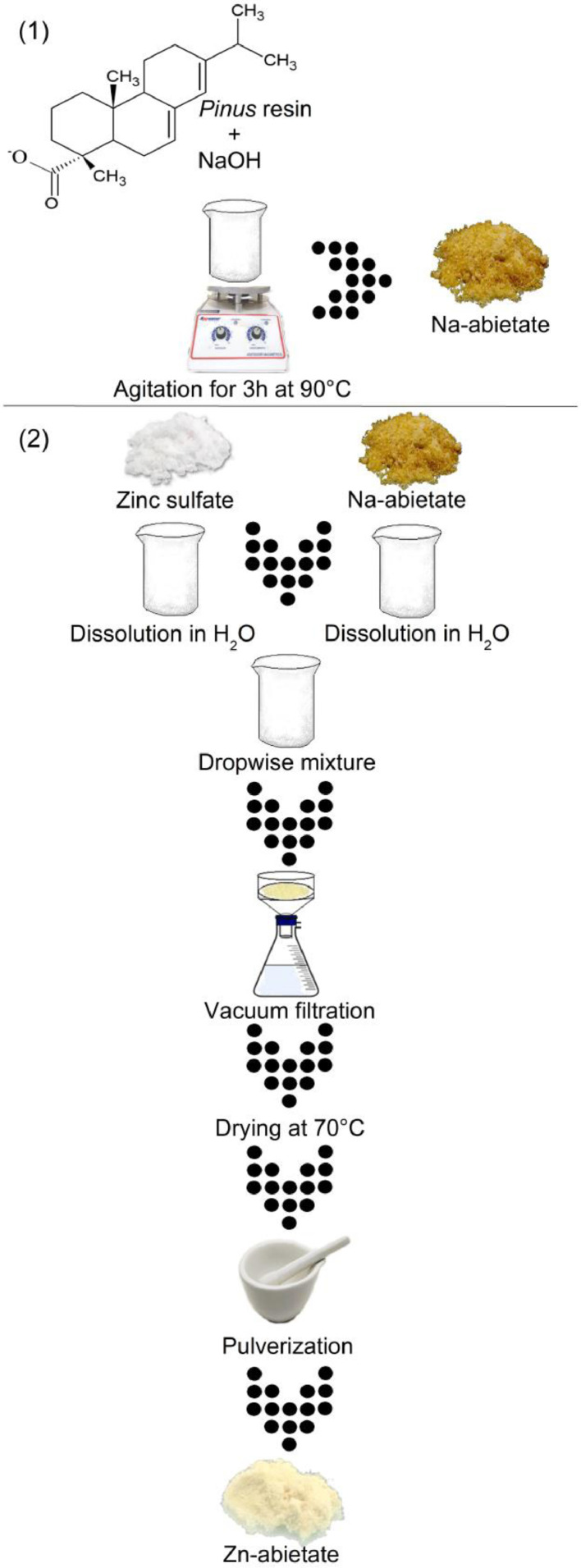
Flowchart of the synthesis methodology of the Zn-abietate complex and its precursor, Na-abietate.

**Fig. 2:**
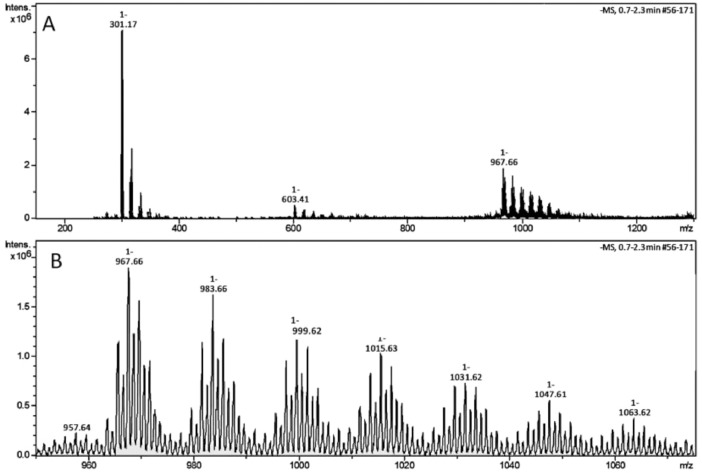
A: Mass spectrum of the Zn-abietate sample. Peaks corresponding to the deprotonated abietic acid [C_20_H_29_O_2_] at m/z 301.17, the formation of a dimeric form of the abietic acid at m/Z 603.41, and the formation of the Zn-abietate complex with three abietate ligands Zn[C_20_H_29_O_2_]^3−^ at m/z 967.66. B: Mass spectrum of the progressive oxidation from the C=C bonds to the corresponding keto-forms of the Zn-abietate complex.

Fig. 1 shows the flowchart of the synthesis methodology for Zn-abietate complex and its precursor, Na-abietate. The main component of *Pinus* resin is the abietic acid with m/z value 301.17 in the Zn-abietate mass spectra (Fig. 2. A). This spectrum shows three main peaks corresponding to m/z of the deprotonated abietic acid [C_20_H_29_O_2_] at 301.17, the formation of a dimeric form of the abietic acid m/z at 603.41, and the formation of the Zn-abietate complex with three abietate ligands Zn[C_20_H_29_O_2_]^3−^ at m/z 967.66, respectively. Fig. 2 B: shows the mass spectrum of the progressive oxidation from the C=C bonds to the corresponding keto-forms (C=O) of the Zn-abietate complex structure.

**Fig. 3:**
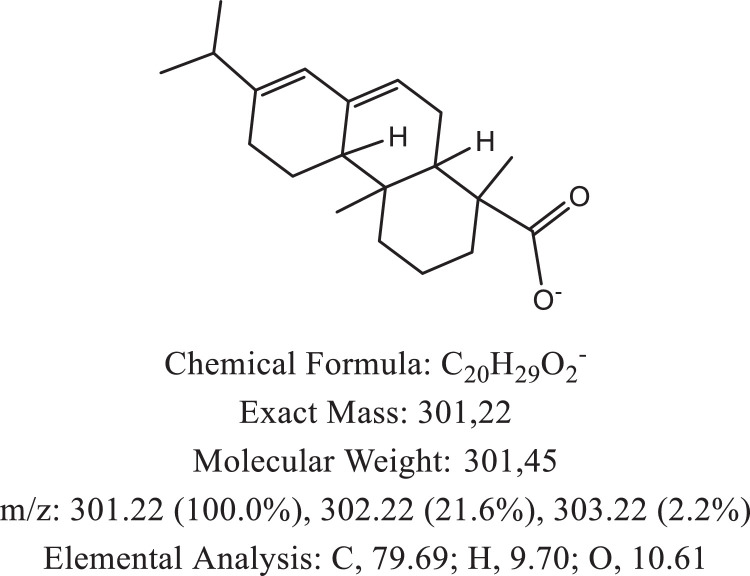
Deprotonated abietic acid structure [C_20_H_29_O_2_].

**Fig. 4:**
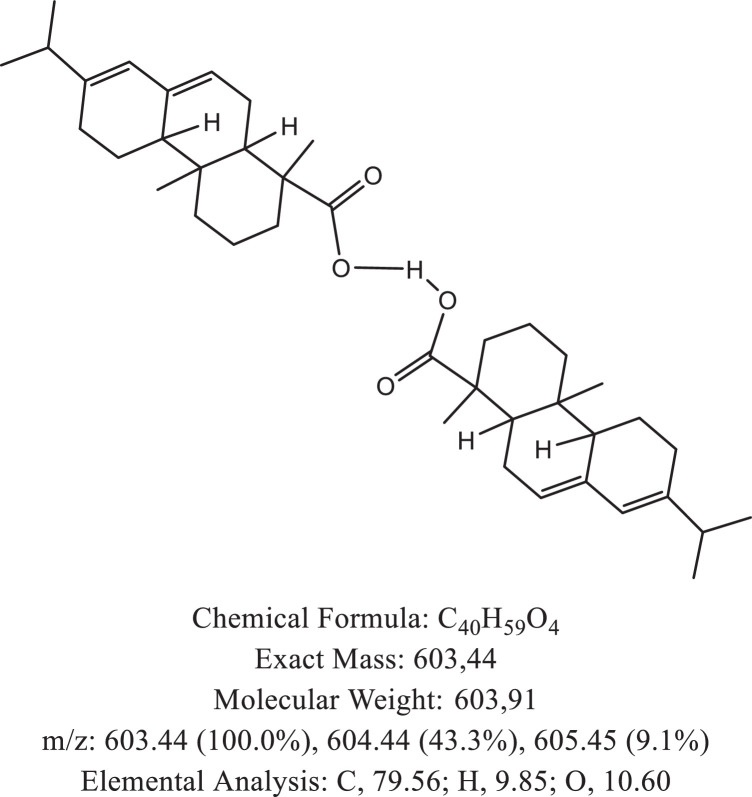
Abietic acid dimeric form structure.

**Fig. 5:**
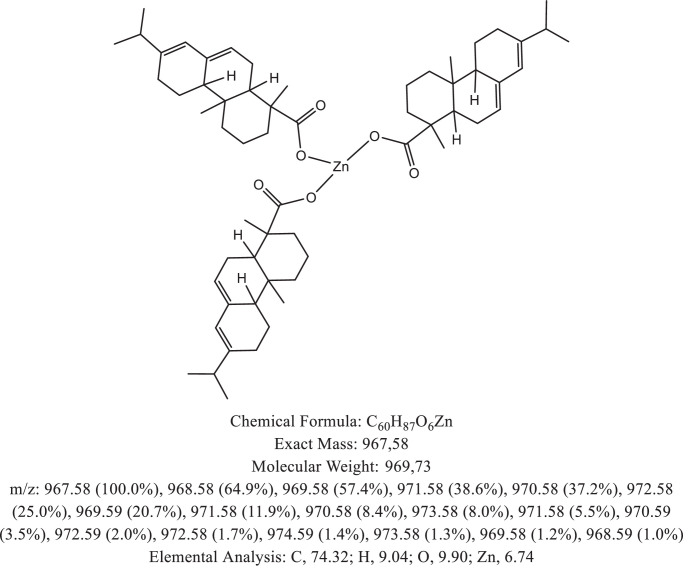
Structural representation of the Zn-abietate complex formed by Zn^2+^ bonded to 3 abietate ligands.

**Fig. 6:**
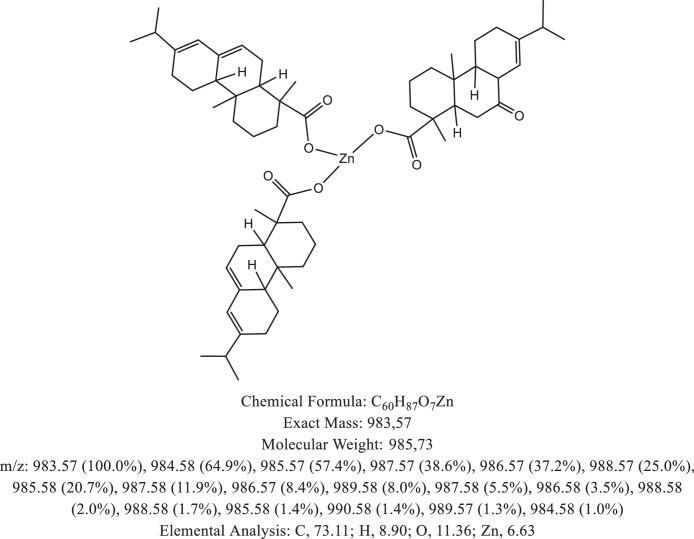
Zn-abietate complex structural representation with one oxidized C = C bond to the keto form.

**Fig. 7:**
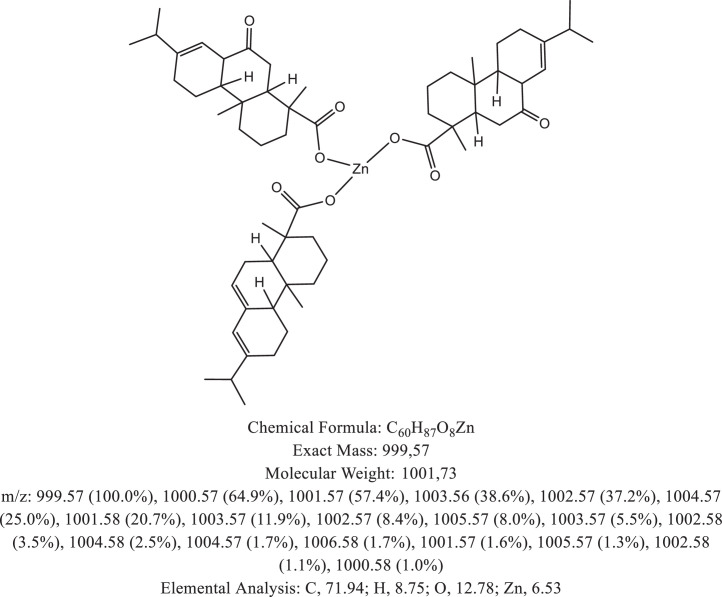
Zn-abietate complex structural representation with two oxidized C = C bonds for the keto form.

**Fig. 8:**
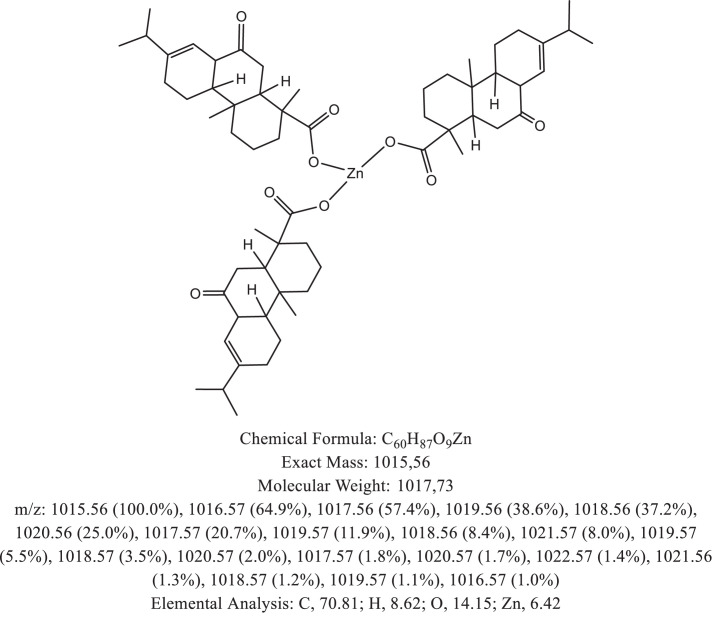
Zn-abietate complex structural representation with three oxidized C = C bonds for the keto form.

**Fig. 9:**
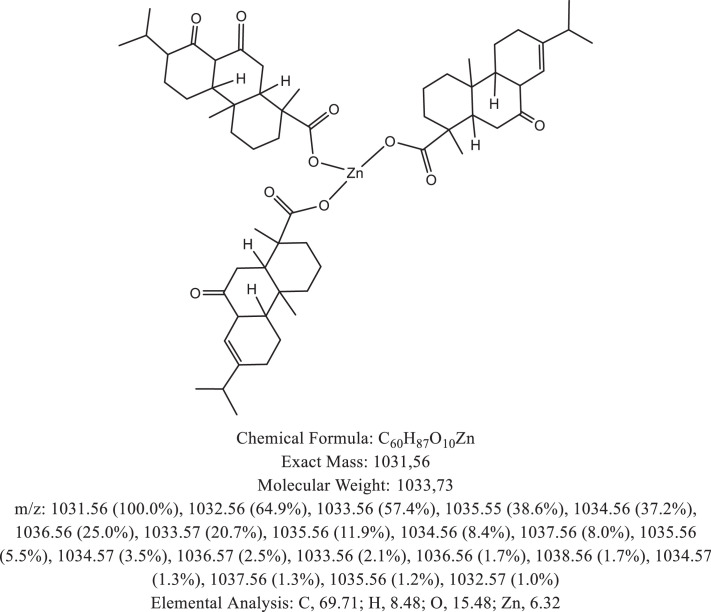
Zn-abietate complex structural representation with four oxidized C = C bonds to the keto form.

**Fig. 10:**
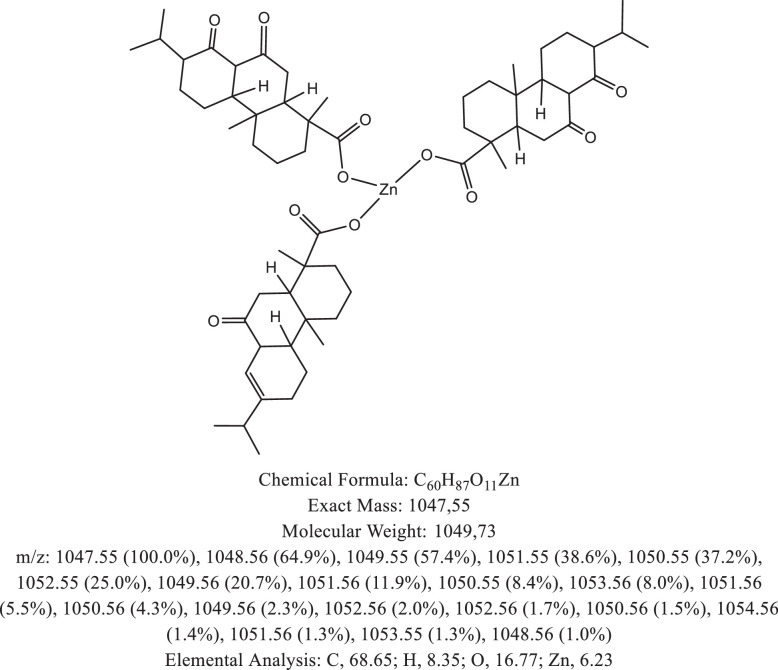
Zn-abietate complex structural representation with five oxidized C = C bonds for the keto form.

**Fig. 11:**
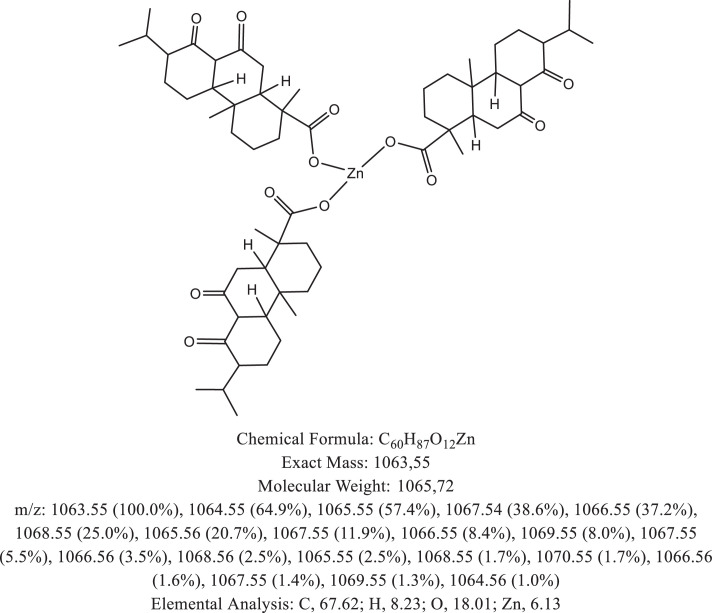
Zn-abietate complex structural representation with six oxidized C = C bonds for the keto form.

The chemical structure of the deprotonated abietic acid corresponding to the peak at m/z 301.17 in Fig. 2A is represented in Fig. 3. Fig. 4 shows the chemical structure of the dimeric form of the abietic acid (peak at m/z 603.41 in Fig. 2A). The structural representation of the Zn-abietate complex with a Zn^2+^ atom bonded to 3 abietate ligands [C_20_H_29_O_2_]^3−^ is showed in Fig. 5, this corresponds to peak at m/z 967.66 in the mass spectrum (Fig. 2A). The chemical structures resulting from the oxidation of the C = C bonds to the keto form are shown in Fig. 6, Fig. 7, Fig. 8, Fig. 9, Fig. 10, Fig. 11, the oxidation process can be observed in the Zn-abietate mass spectrum by a difference of m/z 16 between its consecutive main peaks (Fig. 2B).

**Fig. 12:**
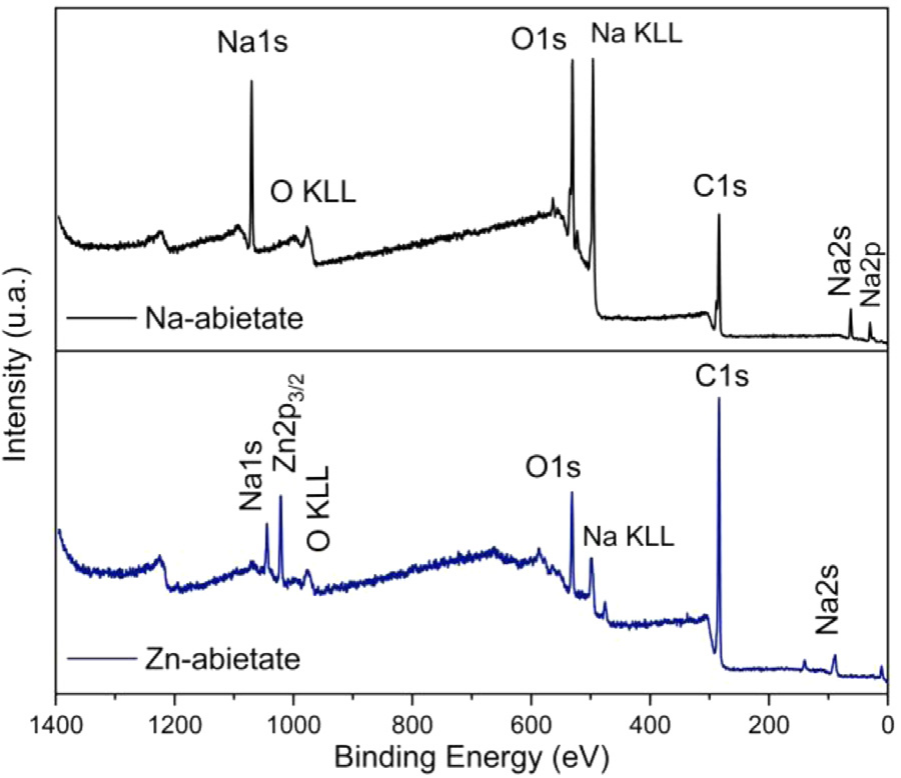
Na-abietate and Zn-abietate XPS Survey spectra.

**Fig. 13:**
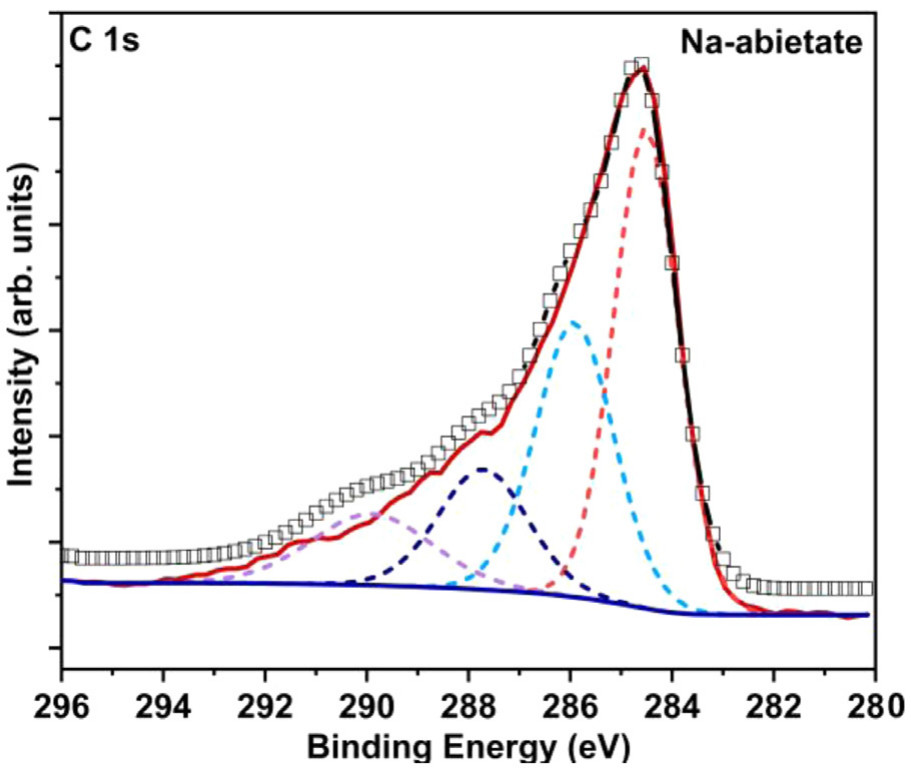
XPS spectra of C1s of the precursor Na-abietate.

**Fig. 14:**
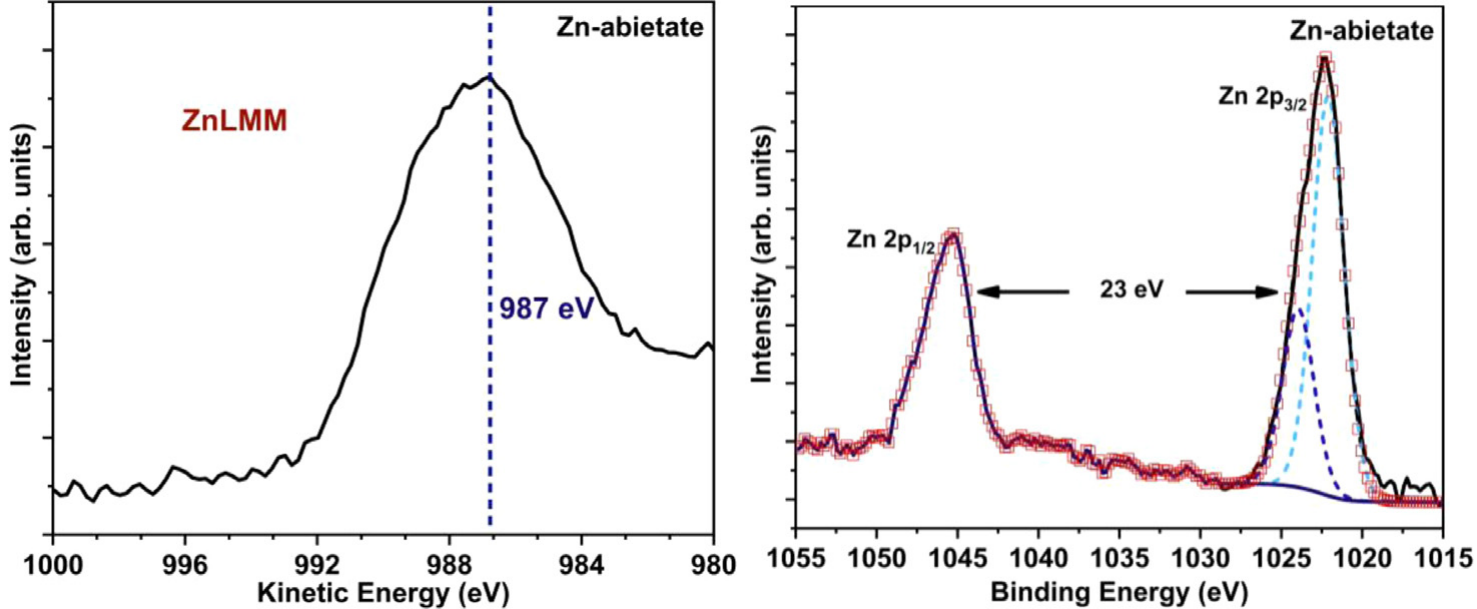
XPS spectra of A: Zn LMM Auger; B: Zn 2p of Zn-abietate sample.

**Fig. 15:**
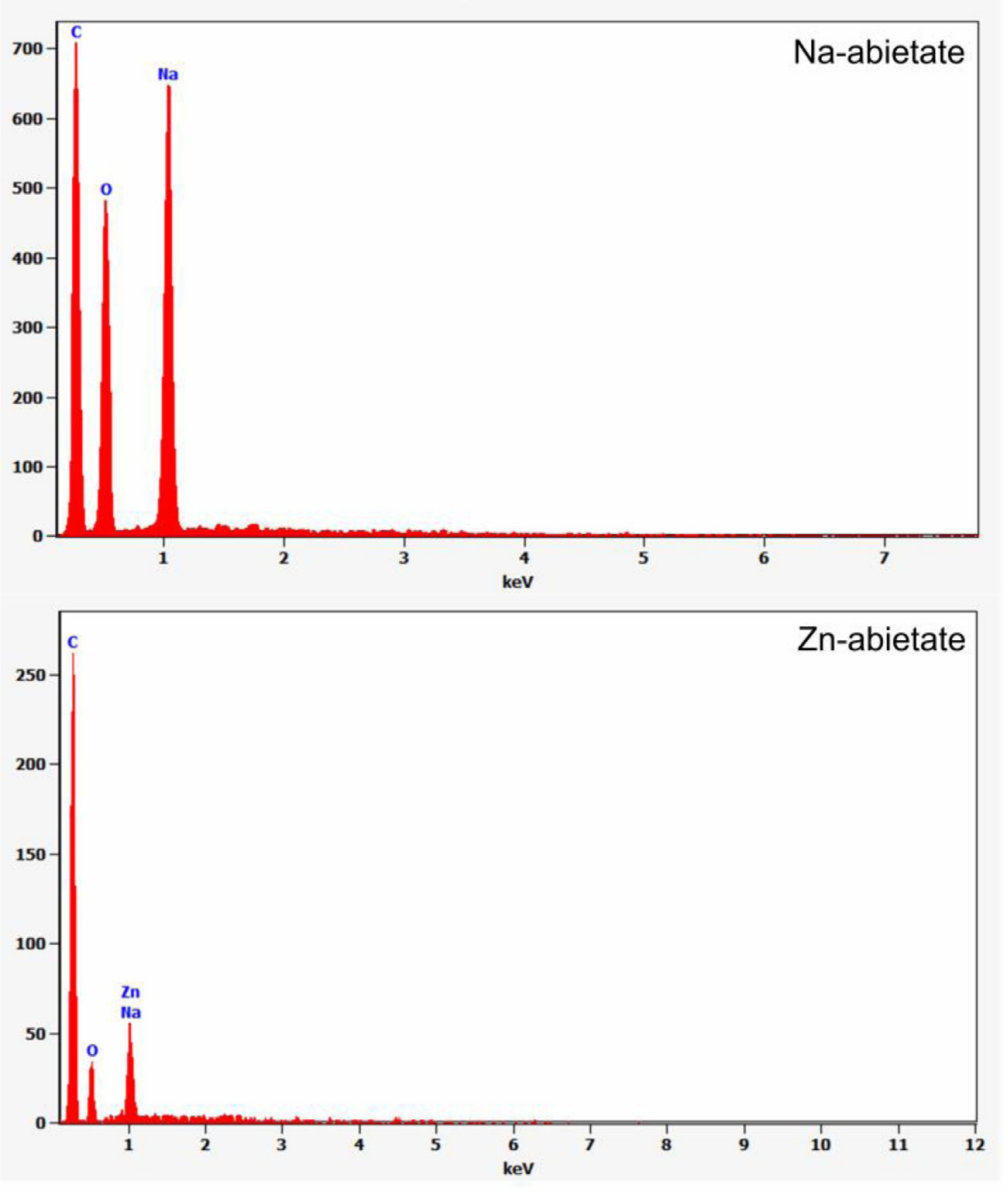
EDS spectra of Na-abietate and Zn-abietate.

The Na-abietate and Zn-abietate XPS survey spectra are presented in Fig. 12, the chemical elements found are labelled. Fig. 13 shows the C 1s spectrum of the Na-abietate. Fig. 14A and 14B show the Zn LMM Auger spectrum and Zn 2p of the Zn-abietate sample, respectively. Energy-dispersive X-ray spectroscopy (EDX) spectrum of Na-abietate and Zn-abietate and the percentages of the elements are presented in Fig. 15, and Table 1, respectively. The raw data of the EDX and XPS analysis found in DOI: 10.17632/n4fhxhz77b.1.

## 2 Experimental Design, Materials and Methods

### 2.1 Synthesis

Fig. 1 describes the Zn-abietate complex synthesis method with its intermediary steps, Na-abietate. *Pinus elliottii* var. *elliottii* resin was used as reaction precursor, this was chosen due to its low cost, abundance, antibacterial properties, and being a regional reforestation product. The reaction has 2 steps as shows the Fig. 1, in the first, 164.0 g of purified *Pinus* resin [1] is mixed with 21.67 g of NaOH dissolved in 200 mL of deionized water; the molar proportion used in this step was 1:1. This mixture is kept under stirring at 90 °C for 3h, all reactional water is evaporated in the course of the reaction for the formation of a hygroscopic salt (Na-abietate). In the second step occurs the Zn-abietate formation from Na-abietate synthetized before; 30.1 g (0.0933 mol) of Na-abietate is dissolved in 50 mL of deionized water, for a faster dissolution the water is heated to 80 °C. This solution is reserved to cooling. A second solution is prepared with 9.0 g (0.0311 mol) of zinc sulfate (ZnSO_4_.7H_2_O, 99%, Quimex) in 30 mL of deionized water. Then, both solutions are mixed dropwise under stirring, and colds in an ice bath. The Zn-abietate formation occurs immediately by the substitution reaction according to Eq. (1). This complex is washed out with deionized water and vacuum filtered, dried in the oven at 70 °C for 5 h, mechanically macerated, and sieved until obtaining the necessary granulometry for application as pigment. The powder-like product shows a coloration of light yellow, and hydrophobic properties The molar proportions for Zn-abietate formation in the second step was 3:1 (Na-abietate:Zn^2+^).

(1) $${{\left[\text{Zn}{\left(OH_{2}\right)}_{6}\right]}^{2+}}_{\left(\text{aq}\right)}+3\;\text{abietat}e_{\left(\text{aq}\right)}\to {{\left[\text{Zn}{\left(\text{abietate}\right)}_{3}\right]}^{2+}}_{\left(\text{aq}\right)}+6H_{2}O_{\left(l\right)}$$

### 2.2 Mass spectrometry

The largest peak in the mass spectrum (Fig. 2) is the ion observed in m/z +301, corresponding to the deprotonated abietic acid theoretical molecular mass [C_20_H_29_O_2_] (Fig. 3). The structure of the abietic acid dimeric form (Fig. 4) corresponding to the peak in m/z +603 in the mass spectrum (Fig. 2). The Zn-abietate complex structural representation formed by Zn^2+^ bonded to 3 abietate ligands [C_20_H_29_O_2_]^3−^ (Fig. 5) is responsible for the peak in m/z +967 in the mass spectrum. The peaks in m/z +983, m/z +999, m/z +1015, m/z +1031, m/z +1047, m/z +1063, correspond to structures responsible in Fig. 6, Fig. 7, Fig. 8, Fig. 9, Fig. 10, Fig. 11. These structures exhibit a characteristic progression with a difference m/z of 16 units for each peak, related to atoms from the oxidation of the C = C bonds to the keto form.

### 2.3 Chemical analysis by X-ray photoelectron spectroscopy (XPS)

The chemical composition was evaluated by X-ray photoelectron spectroscopy (XPS). All binding energies were calibrated using the carbon C 1s peak at 284.6 eV. The elemental analysis of the Zn-abietate complex and Na-abietate salt was performed using XPS survey scans. The elements detected in samples were C, O, and Na for Na-abietate; and C, O, Na, and Zn for Zn-abietate. XPS survey scan revealed lower contamination with Na in the Zn-abietate sample. The sodium observed in the survey spectrum can be associated with remaining residues from the first synthesis stage.

Na-abietate precursor X-ray photoelectron analysis revealed the presence of carbon (51.6 at%), oxygen (30.8 at%), sodium (16.6 at%), and sulfur (1.0 at%). The spectrum of O 1s for the Na-abietate can be reproduced using five components (Gaussians-Lorentzian) centered at 531.2 eV, 533.8 eV, 535.3 eV, 536.8 eV, and 532.6 eV corresponding to the Na-O, to the carbonyl group, and/or carbon-oxygen atoms simple bonds (O-C/O=C), carboxylate group oxygen atoms (O-C=O) [2], Na KL_1_L_23_ Auger emission, and sulfate (SO_4_)^2−^ [3], respectively. The C 1s spectrum of Na-abietate (Fig. 13) shows components centred at binding energies of 284.5 eV, 285.9 eV, 287.7 eV, 289.9 eV, 289.9 eV, assigned, respectively, to sp^3^ carbon bonds to hydrogen atoms or other carbon (C-H/C-C), C-O bonds, and/or carbonyl group (C=O bonds), carboxylate group O-C-O, and O-C=O bonds [2]. The XPS analysis can be used to determine the coordinating metal atom oxidation state. This is important information to understand metals ion antibacterial properties as in the case of zinc [4,5]. For the Zn-abietate sample, the Zn LMM Auger peak centred at 987 eV indicates that the Zn atoms have the Zn (II) oxidation state (Fig. 14A). In the XPS spectrum the distance between Zn 2p_3/2,_and 1p_1/2_ is 23 eV (Fig. 14B) confirming the Zn^2+^ oxidation state.

### 2.4 Chemical analysis by EDS

The energy-dispersive X-ray spectroscopy (EDS) analysis was performed in quadruplicate. Fig. 15 shows the curves for the elements, and the percentages are summarized in Table 1. In the Zn-abietate sample was detectable contamination sodium due to the Na-abietate formed in the first stage of synthesis. For the Na-abietate no contaminants were identified by this technique due to the low sensitivity to surface contamination of this method.

**Table 1:** Energy-dispersive X-ray spectroscopy (EDX) quantitative results for Na-abietate and Zn-abietate.

Element	Na-abietate		Zn-abietate	
	Weight %	Atom %	Weight %	Atom %
Carbon	25.5 ± 0.4	34.1 ± 1.0	57.2 ± 1.0	76.0 ± 2.6
Oxygen	40.3 ± 0.6	41.3 ± 1.2	17.9 ± 1.2	17.9 ± 2.4
Sodium	34.2 ± 0.4	24.6 ± 0.5	0.2 ± 0.3	0.1 ± 0.4
Zinc	-	-	24.7 ± 1.1	6.0 ± 0.5
Total	100	100	100	100

## CRediT authorship contribution statement

**Jamille S. Correa:** Conceptualization, Methodology, Validation, Formal analysis, Investigation, Writing – original draft. **Júlia O. Primo:** Formal analysis, Investigation. **Carla Bittencourt:** Investigation, Writing – review & editing, Supervision. **Dienifer F.L. Horsth:** Formal analysis, Investigation. **Eduardo Radovanovic:** Formal analysis, Investigation. **Alceu T. Silveira-Jr:** Validation, Formal analysis. **Henrique E. Toma:** Writing – review & editing, Supervision. **Cristina M. Zanette:** Methodology, Writing – review & editing, Supervision. **Fauze J. Anaissi:** Conceptualization, Writing – review & editing, Supervision, Project administration.

## Declaration of Competing Interest

The authors declare that they have no known competing financial interests or personal relationships that could have appeared to influence the work reported in this paper.
